# Whole-Genome Sequencing Reveals Temporal Trends in Antibiotic Resistance Genes in *Escherichia coli* Causing Pediatric Urinary Tract Infections in Central Vietnam

**DOI:** 10.3390/antibiotics13090830

**Published:** 2024-09-02

**Authors:** Huyen Thanh Thi Le, Trang Thu Hoang, Ngoc Anh Thi Nguyen, Sang Ngoc Nguyen, Ung Dinh Nguyen, Cuong Xuan Hoang, Nam S. Vo, Duc Quang Le, Son Hoang Nguyen, Minh Duc Cao, Tho Huu Ho

**Affiliations:** 1 Department of Pediatrics, Faculty of Clinical Internal Medicine, Vinh Medical University, Vinh 431000, Vietnam; lehuyenvinh@gmail.com; 2 Department of Genomics, Institute of Biomedicine & Pharmacy (IBP), Vietnam Military Medical University, Hanoi 10000, Vietnam; hoangtrang8a1@gmail.com (T.T.H.); anhnguyen.011188@gmail.com (N.A.T.N.); dr.ungd4.vmmu@gmail.com (U.D.N.); 3 Pediatric Department, Haiphong University of Medicine and Pharmacy, Haiphong 04254, Vietnam; nnsang@hpmu.edu.vn; 4 Department of Military Science, Vietnam Military Medical University, Hanoi 10000, Vietnam; hoangxuancuong@vmmu.edu.vn; 5 Center for Biomedical Informatics, Vingroup Big Data Institute, Hanoi 10000, Vietnam; v.namvs@vinbigdata.org; 6 Faculty of IT, National University of Civil Engineering, Hanoi 11616, Vietnam; leducquangpm@gmail.com; 7 Amromics JSC, Vinh 431000, Vietnam; dr.sonhoangnguyen@gmail.com (S.H.N.); minhduc.cao@gmail.com (M.D.C.); 8 Department of Microbiology, Vietnam Military Medical University, Hanoi 10000, Vietnam

**Keywords:** pediatric UTIs, *Escherichia coli*, antibiotic resistance, genetic mechanisms, whole-genome sequencing, temporal trends, central Vietnam

## Abstract

(1) Background: Pediatric urinary tract infections (UTIs) pose significant challenges due to drug-resistant *Escherichia coli* (*E. coli*) strains. This study utilizes whole-genome sequencing to analyze temporal trends in antibiotic resistance genes (ARGs) in clinical *E. coli* isolates from pediatric UTI cases in central Vietnam. (2) Methods: We conducted whole-genome sequencing on 71 *E. coli* isolates collected from pediatric UTI patients between 2018 and 2020. ARGs were identified, and their prevalence over time was analyzed. Statistical tests were used to correlate ARG presence with antibiotic resistance. (3) Results: Of the 47 *E. coli* isolates with complete data, 40 distinct ARGs were identified, with a median of 10 resistance genes per isolate. A significant increase in the total number of ARGs per isolate was observed over time, from an average of 8.88 before June 2019 to 11.63 after. Notably, the prevalence of the *aadA2* gene (aminoglycoside resistance) rose from 0% to 26.7%, and that of the *blaNDM-5* gene (beta-lactam and carbapenem resistance) increased from 0% to 23.3%. Key correlations include blaEC with cephalosporin resistance, *blaNDM-5* with carbapenem resistance, and *sul2* with sulfamethoxazole/trimethoprim resistance. (4) Conclusions: Whole-genome sequencing reveals complex and evolving antibiotic resistance patterns in pediatric *E. coli* UTIs in central Vietnam, with a marked increase in ARG prevalence over time. Continuous surveillance and targeted treatments are essential to address these trends. Understanding genetic foundations is crucial for effective intervention strategies.

## 1. Introduction

Urinary tract infections (UTIs) are a prevalent concern in pediatric healthcare, affecting a significant number of children, particularly girls, with up to 3% of girls and 1% of boys experiencing at least one UTI during prepubertal age [1]. Among the causative agents, *Escherichia coli* (*E. coli*) bacteria are the predominant culprits in pediatric UTIs [1]. Several risk factors, such as female sex, structural urinary tract abnormalities, vesicoureteral reflux, and others, contribute to children’s susceptibility to UTIs [1,2]. Untreated UTIs can lead to severe complications, including kidney scarring, renal failure, and long-term kidney damage [1]. Thus, timely diagnosis and treatment are crucial to mitigate these complications [1]. Preventive measures, including hygiene practices and the use of prophylactic antibiotics in high-risk children, play an essential role in reducing the UTI burden in children.

The rise of drug-resistant *E. coli* strains that cause UTIs in children presents an escalating challenge [3]. Over recent years, the prevalence of drug-resistant *E. coli* strains has increased, primarily due to the overuse and misuse of antibiotics [3]. Such drug-resistant infections can result in treatment failures, prolonged hospital stays, and higher healthcare costs [4]. Identifying drug-resistant strains is pivotal for selecting appropriate antibiotics for the management of UTIs in children. Reducing unnecessary antibiotic use, promoting hygiene practices, and implementing antibiotic stewardship programs are critical for mitigating the emergence and spread of drug-resistant *E. coli* strains [5].

Drug resistance in *E. coli* strains that cause pediatric UTIs often has a genetic basis. Specific genes and genetic mutations have been pinpointed as contributors to drug resistance, encoding antibiotic efflux pumps, enzymes for antibiotic modification or degradation, or alterations in bacterial targets [6]. Additionally, horizontal gene transfer, facilitated by plasmids, amplifies drug resistance in *E. coli* strains [7]. Whole-genome sequencing (WGS) has substantially enhanced our understanding of these mechanisms [8]. WGS allows for the identification of specific genetic mutations, characterizes *E. coli* strains, tracks their transmission, and aids in developing diagnostic tools [8,9,10]. The integration of sequencing data with clinical and epidemiological information provides invaluable insights into the spread and persistence of drug-resistant strains, paving the way for targeted interventions [9].

While antibiotics remain the primary treatment for pediatric UTIs caused by drug-resistant *E. coli*, the increasing prevalence of resistance limits antibiotic choices [1]. Trimethoprim/sulfamethoxazole (TMP-SMX) is the first-line treatment for many *E. coli* strains, alongside other antibiotics like nitrofurantoin, amoxicillin-clavulanate, and cephalexin [11,12]. However, resistance to these antibiotics has grown, necessitating alternative treatment options such as fosfomycin, ciprofloxacin, and amikacin. The selection of antibiotics should be guided by bacterial culture and susceptibility testing to ensure the most effective treatment [12].

Therefore, the rising prevalence of drug-resistant *E. coli* strains causing pediatric UTIs underscores the need for effective strategies for prevention and management. Leveraging whole-genome sequencing to understand genetic mechanisms, integrating epidemiological insights, and judiciously selecting antibiotics are crucial steps toward mitigating this public health challenge. This study aims to analyze the temporal trends in the antibiotic resistance genes in *E. coli* causing pediatric UTIs in central Vietnam, using whole-genome sequencing to provide a comprehensive genetic characterization and identify evolving resistance patterns.

## 2. Results and Discussion

### 2.1. Epidemiological and Microbiological Profiling of Clinical E. coli Isolates

To establish a foundational understanding of the epidemiology and microbiology of pediatric urinary tract infections (UTIs) caused by *E. coli* in Central Vietnam, we conducted a comprehensive investigation of clinical isolates from pediatric patients (Table 1). Our study revealed a high prevalence of *E. coli* as the causative agent in pediatric UTIs, accounting for 83.7% of the cases. This significant finding underscores the predominant role of *E. coli* in UTIs among children in this region. The gender distribution among affected children was nearly equal, with a slight preponderance of females (51.0%) over males (49.0%). Age distribution analysis showed that the majority of UTI cases (28.6%) occurred in children aged 2 months to under 1 year, highlighting the vulnerability of younger children to *E. coli* infections.

A noteworthy urban–rural discrepancy was observed, with a substantial proportion (70.4%) of cases originating from rural areas compared to urban areas (29.6%). This disparity raises important questions about the potential influence of environmental and socio-economic factors on the prevalence of UTIs in pediatric populations [13]. Further research into these factors could provide valuable insights into targeted preventive measures.

The clinical presentations of the patients were diverse, with the primary reasons for hospital admission being fever, urinary symptoms, and abdominal pain. Additionally, the patients’ documentation revealed the presence of external genitalia abnormalities in some cases, which may contribute to their susceptibility to UTIs. Hematological and urinary abnormalities, including elevated white blood cell counts, neutrophil counts, and C-reactive protein levels, were common, indicating the severity of the infections. Ultrasonography findings complemented these observations by detecting renal and bladder abnormalities in certain cases, providing a comprehensive picture of the clinical impact of UTIs on pediatric patients.

A microbiological analysis of the clinical *E. coli* isolates revealed several critical insights. The identification of extended-spectrum beta-lactamase (ESBL)-producing *E. coli* isolates highlights the growing concern about antibiotic resistance in UTIs, emphasizing the need for prudent antibiotic use. High resistance rates to ampicillin (98.2%), sulfamethoxazole/trimethoprim (82.9%), and cephalosporin (68.6%) were observed among these isolates, indicating the limited effectiveness of these commonly used antibiotics for UTI treatment in the region. Additionally, variable resistance patterns were detected for other antibiotics, underscoring the complexity of antibiotic resistance in clinical *E. coli* isolates from pediatric UTI cases.

The high prevalence and significant antibiotic resistance observed necessitate further genetic analysis to understand the mechanisms driving this resistance.

### 2.2. Identification of Antibiotic Resistance-Conferring Genes

Building on the epidemiological and microbiological profiling, our study proceeded to identify the specific antibiotic resistance-conferring genes present in the *E. coli* isolates from pediatric UTI cases. This genetic analysis aimed to elucidate the underlying mechanisms of antibiotic resistance, providing crucial insights into the genetic basis of resistance and informing targeted treatment strategies.

**Figure 1:**
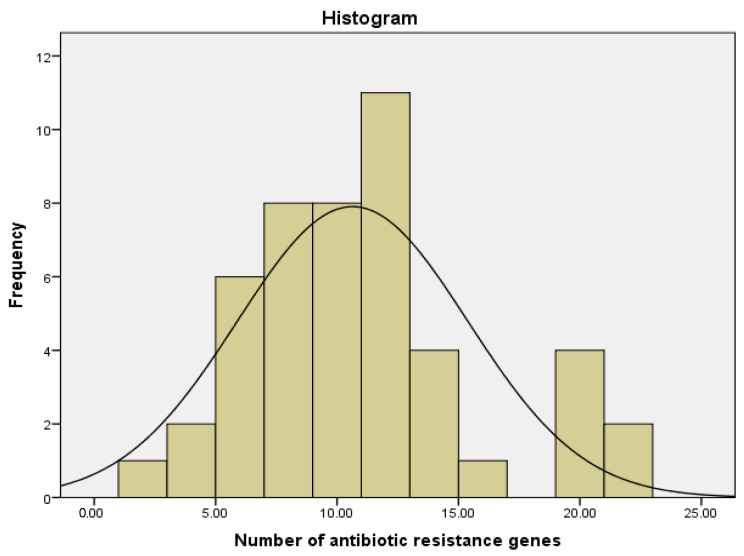
Number of antibiotic resistance genes in isolated *E. coli* samples. This figure illustrates the distribution of antibiotic resistance genes in isolated *E. coli* samples, indicating the median of 10 genes per sample and the interquartile range from 7 to 12 genes per sample.

A comprehensive genetic analysis was conducted on 71 *E. coli* isolates, with 47 samples yielding complete data. Our findings revealed a wide distribution of antibiotic resistance genes (ARGs) among these isolates. The median number of resistance genes per isolate was 10, with an interquartile range from 7 to 12 genes, indicating significant variability in the genetic makeup of the isolates (Figure 1). This diversity of ARGs underscores the complexity of antibiotic resistance mechanisms in the *E. coli* population studied [14,15].

Within our cohort of 47 isolated *E. coli* strains, we unveiled a spectrum of 40 distinct antibiotic resistance genes (ARGs) that underscore the intricate landscape of antibiotic resistance (Table 2). These ARGs can be categorized based on their resistance to various antibiotic classes, shedding light on the multifaceted challenge of combating resistance in urinary tract infections (UTIs) caused by *E. coli* in pediatric patients. 

Intriguingly, 11 ARGs were associated with beta-lactam resistance, reflecting the potential hurdles in treating *E. coli* infections within this cohort. The aminoglycoside resistance genes, numbering 11, further emphasize the importance of prudent antibiotic use. Additionally, eight ARGs were linked to trimethoprim/sulfamethoxazole resistance, a commonly employed antibiotic. Three ARGs conferred resistance to quinolone antibiotics, which are vital in clinical practice. Furthermore, nine other ARGs were detected, signifying resistance to other antibiotic classes.

The implications of these findings are profound. The presence of resistance genes against essential antibiotics not only limits treatment options but also emphasizes the urgency of enhanced antibiotic stewardship practices [5]. The intricate distribution of resistance genes across diverse antibiotic classes highlights the complex mechanisms driving antibiotic resistance within these *E. coli* isolates [7]. Addressing this challenge requires a multifaceted approach encompassing genetics, clinical strategies, and public health interventions.

Given the wide distribution of antibiotic resistance genes among *E. coli* isolates, it is crucial to investigate how these genes’ prevalence changes over time to identify emerging resistance trends.

### 2.3. Correlation of Antibiotic Resistance Genes and Sample Collection Time

In order to understand the temporal dynamics of antibiotic resistance genes (ARGs) within the *E. coli* isolates, we conducted a detailed analysis of the relationship between the presence of specific ARGs and the time of sample collection. This section aims to identify trends and shifts in the prevalence of resistance genes over the course of the study period, providing insights into the evolving landscape of antibiotic resistance.

Our analysis spanned approximately 28 months, using the midpoint of the sample collection period (1 June 2019) as a reference. We observed a significant increase in the average number of ARGs over this period. Specifically, the average number of ARGs before 1 June 2019 was 8.88 (95% CI: 7.81, 9.95), while it was 11.63 (95% CI: 9.86, 13.41) after 1 June 2019. A more detailed analysis reveals a significant difference in the standard deviation between the two time groups, with the standard deviation of the post-June 1, 2019 group being 5.24 compared to 3.12 for the pre-June 1, 2019 group. This indicates a higher degree of variability in the number of ARGs after 1 June 2019. The statistical tests faced challenges due to the nature and differences in data distribution, but confidence intervals demonstrate a clear trend of increasing ARG numbers after 1 June 2019.

In addition to the overall increase in ARGs, we observed several statistically significant changes in the frequency of specific ARGs over this period (*p* < 0.05, Fisher’s exact test). Notably, the following genes exhibited substantial increases in prevalence:

- *aadA2* (Aminoglycoside resistance): The *aadA2* gene, associated with resistance to aminoglycosides, showed a significant increase in frequency, rising from 0% (0/17) in the early part of the study to 26.7% (8/30) in the latter period. This trend suggests an increasing difficulty in treating infections with aminoglycosides over time.
- *blaNDM-5* (Beta-lactam and carbapenem resistance): The *blaNDM-5* gene, responsible for conferring resistance to beta-lactam antibiotics, including carbapenems, exhibited a substantial increase from 0% (0/17) to 23.3% (7/30). This alarming trend underscores the critical need for monitoring and addressing carbapenem resistance in clinical settings.
- *ble* (Bleomycin resistance): The *ble* gene, associated with bleomycin resistance, also demonstrated a significant rise in prevalence, from 0% (0/17) to 23.3% (7/30). Given the clinical relevance of bleomycin as an antitumor antibiotic, this trend highlights the importance of continued surveillance and research into resistance mechanisms.
- *dfrA12* (Trimethoprim resistance): The *dfrA12* gene, linked to trimethoprim resistance, showed an increase from 0% (0/17) to 26.7% (8/30). This shift indicates a potential change in resistance patterns to this commonly used antibiotic.

The observed temporal trends in the prevalence of specific ARGs emphasize the dynamic nature of antibiotic resistance in *E. coli* isolates from pediatric UTI cases. The increase in the frequency of these genes over time highlights the need for continuous surveillance and adaptive strategies in antibiotic stewardship. Understanding these shifts is crucial for developing effective interventions and mitigating the impact of antibiotic resistance on clinical outcomes.

Continuous monitoring and further research are warranted to confirm these findings and explain the underlying factors driving this increase. Addressing this potential rise in antibiotic resistance is crucial for developing effective treatment strategies and public health interventions to manage pediatric urinary tract infections (UTIs) effectively.

The temporal analysis of antibiotic resistance genes provides valuable insights into the evolving patterns of resistance within the *E. coli* isolates. The significant increases in the prevalence of genes such as *aadA2*, *blaNDM-5*, *ble,* and *dfrA12* indicate emerging resistance challenges that require immediate attention and ongoing monitoring. These findings underscore the importance of integrating genetic analysis with epidemiological data to inform targeted treatment approaches and public health strategies.

### 2.4. Correlation of Antibiotic Resistance Genes and Antibiotic Susceptibility Profiles

To provide a comprehensive understanding of the clinical implications of antibiotic resistance genes (ARGs) in *E. coli* isolates, we examined the correlation between the presence of specific ARGs and antibiotic susceptibility profiles. This analysis aimed to elucidate how genetic resistance factors translate into phenotypic resistance, thereby informing more precise and effective treatment strategies for pediatric UTIs.

#### 2.4.1. Beta-Lactam Resistance Genes and Antibiotic Susceptibility

We investigated the relationship between various beta-lactam resistance genes (e.g., *blaEC*, *blaTEM-1*, *blaCTX-M-27*, *blaNDM-5*) and the resistance profiles of third-generation cephalosporins and carbapenems. Our analysis revealed that the presence of the blaEC gene was significantly correlated with cephalosporin resistance (*p* = 0.041), indicating its crucial role in conferring resistance to this class of antibiotics. Similarly, the *blaNDM-5* gene exhibited a strong association with carbapenem resistance (*p* = 0.001), highlighting the critical need to monitor this gene to manage effective treatment options in clinical settings [16]. These findings emphasize the importance of specific beta-lactam resistance genes in contributing to resistance profiles, underscoring the necessity for comprehensive susceptibility testing to guide appropriate antibiotic use.

#### 2.4.2. Amikacin Resistance and Aminoglycoside Resistance Genes

Our study further explored the correlation between amikacin resistance and several aminoglycoside resistance genes (e.g., aac(6′)-Ib-cr5, *aadA2*, *rmtB1*, *aadA16*). The analysis demonstrated significant associations between the presence of these genes and amikacin resistance. Specifically, aac(6′)-Ib-cr5 (*p* = 0.034), *aadA2* (*p* = 0.034), *rmtB1* (*p* = 0.018), and *aadA16* (*p* = 0.007) were all strongly linked to amikacin resistance. These correlations suggest that the presence of these genes is closely related to amikacin resistance in the *E. coli* isolates, underscoring the complexity of antibiotic resistance mechanisms and the importance of targeted susceptibility testing. Understanding these associations is crucial for clinical management, as amikacin is a critical antibiotic used to treat severe infections [12].

The emergence of amikacin resistance, particularly in conjunction with resistance to other commonly used antibiotics, poses a significant challenge in clinical practice. Amikacin often serves as a last-resort treatment for multidrug-resistant infections when other antibiotics fail [17]. Therefore, the presence of amikacin resistance genes and their associations with aminoglycoside resistance genes underscore the growing complexity of antibiotic resistance in clinical settings.

#### 2.4.3. Sulfamethoxazole/Trimethoprim Resistance and Relevant Genes

We also examined the relationship between sulfamethoxazole/trimethoprim resistance and relevant resistance genes (e.g., *sul2*, *sul1, dfrA17*, *dfrA12*). Among these genes, *sul2* showed a significant correlation with resistance to sulfamethoxazole/trimethoprim (*p* = 0.001). Notably, 93.3% (28/30) of samples harboring the *sul2* gene exhibited resistance to sulfamethoxazole/trimethoprim, while 50% (8/16) of samples lacking this gene were susceptible to the antibiotic combination. These results highlight the pivotal role of the *sul2* gene in mediating resistance to sulfamethoxazole/trimethoprim, emphasizing the need for careful consideration of resistance patterns when selecting treatment regimens.

These findings underscore the pivotal role of *sul2* in conferring resistance to sulfamethoxazole/trimethoprim in our sampled *E. coli* strains. The high prevalence of *sul2* among the resistant strains highlights its importance as a key determinant of resistance to this antibiotic combination.

Sulfamethoxazole/trimethoprim is commonly used to treat a range of bacterial infections, making its efficacy crucial in clinical practice [18]. However, the emergence of resistance, particularly associated with genes like *sul2*, poses challenges to treatment outcomes. It is essential for clinicians to consider the presence of such resistance genes when prescribing sulfamethoxazole/trimethoprim and to explore alternative treatment options when necessary.

The correlations identified between specific ARGs and antibiotic susceptibility profiles provide critical insights into the genetic foundations of antibiotic resistance in pediatric *E. coli* isolates. While the presence of these genes is significantly associated with resistance, it is important to note that the presence of resistance genes does not always equate to phenotypic resistance. This indicates the need for further research to explore environmental and regulatory factors influencing gene expression and resistance phenotypes.

Understanding the genetic basis of antibiotic resistance and its correlation with phenotypic resistance is essential for developing targeted treatment strategies. These findings can inform antibiotic stewardship programs and improve clinical outcomes by tailoring treatments based on genetic resistance profiles. Ongoing surveillance and comprehensive genetic analyses are crucial to adapt to the evolving landscape of antibiotic resistance, ultimately contributing to global efforts in combating this critical public health issue. By integrating genetic and phenotypic data, our study enhances the understanding of antibiotic resistance mechanisms and supports the development of effective interventions to manage antibiotic-resistant UTIs in pediatric populations.

## 3. Materials and Methods

### 3.1. Study Design and Setting

The research was conducted from 2018 to 2020 at Nghe An’s Obstetrics and Pediatrics Hospital, with the genetic analysis of bacterial specimens carried out at the Department of Genomics, Institute of Biomedicine & Pharmacy, Vietnam Military Medical University. The study was ethically approved and adhered to the principles of the Helsinki Declaration.

### 3.2. Study Participants

The study encompassed 392 pediatric patients aged 2 months to 15 years who sought medical care for fever at Nghe An’s Obstetrics and Pediatrics Hospital between 2018 and 2020. These individuals were both diagnosed with and received treatment for febrile urinary tract infection (UTI) at the same medical facility during this specific period.

Diagnostic criteria for febrile UTI adhered to National Institute for Health and Clinical Excellence (NICE) guidelines [19]. Exclusion criteria were applied to patients diagnosed with febrile UTI who were also experiencing severe illnesses necessitating intensive care or life-threatening conditions. Additionally, patients with concurrent medical conditions, those who had received outpatient or lower-level antibiotic treatment during the current illness, patients who met the diagnostic criteria but whose families were uncooperative, and individuals with polymicrobial urine cultures were excluded from the study.

### 3.3. Urine Culture Technique for Bacterial Identification

In this study, a urine culture technique was employed to identify bacteria. It involved urine microscopy to detect leukocyturia, indicating a urinary tract infection (UTI). Bacterial isolation followed, and the urine samples were cultured and examined for colony-forming units (CFU). Samples with ≥10^5^ CFU/mL were considered positive.

To briefly describe the isolation process, urine samples were inoculated directly onto Blood agar (BA) (catalog number: TM0722, LABone, Ho Chi Minh, Vietnam) and UTI agar (catalog number: P901520, Melab, Vinh Yen, Vinh Phuc, Vietnam) plates using a calibrated loop. The plates were incubated at 37 °C for 24–48 h. After incubation, colonies were counted to determine CFU/mL, and colonies with different morphologies were isolated for further identification.

### 3.4. Bacterial Identification and Antibiotic Susceptibility Testing

The VITEK^®^2 Compact system (bioMérieux, Craponne, France) was used for bacterial identification and antibiotic susceptibility testing. It utilized colorimetric methods to identify bacteria and perform extended-spectrum beta-lactamase (ESBL) detection. Antibiotic susceptibility testing was carried out to determine the minimum inhibitory concentration (MIC) for each antibiotic.

A loopful of colonies was transferred to 3 mL of sterile saline solution (0.45%) to achieve a turbidity of 0.5–0.63 McFarland. Then, 145 µL of this bacterial suspension was transferred into a tube containing 3 mL of saline. The VITEK^®^2 AST-GN (catalog number: 21341, bioMérieux, France) cards were loaded with the bacterial suspensions and placed into the VITEK^®^2 Compact system for incubation and analysis. Information about each test was entered into the VITEK^®^2 system, including patient details and test specifics. The system automatically processed the cards and provided results, which were reviewed and printed.

The study adhered to the Clinical and Laboratory Standards Institute (CLSI) guidelines for antibiotic susceptibility testing, ensuring the accuracy and reliability of the results.

### 3.5. Bacterial Strain Collection and Preservation

Bacterial strains demonstrating antibiotic resistance, especially *E. coli*, were collected and preserved in BHI medium (catalog number: T510704, Melab, Vinh Phuc, Vietnam) with 20% glycerol at −80 °C for further analysis in the Department of Genomics, Institute of Biomedicine & Pharmacy, Vietnam Military Medical University.

### 3.6. Next-Generation Sequencing

Following bacterial isolation, identification, and antibiotic susceptibility testing, total DNA was extracted from the isolated strains. A commercially available QIAamp DNA Mini Kit (catalog number: 51306, Qiagen, Hilden, Germany) was employed to extract DNA. The procedure included several steps, such as cell lysis, proteinase digestion, binding of DNA to a column, washing, and elution. After extraction, the DNA was preserved at −20 °C until further use. DNA concentration was quantified using a Qubit fluorometer (Thermo Fisher Scientific, Waltham, MA, USA), and normalization was performed to prepare the samples for genomic sequencing. The DNA was fragmented into 300–500 nucleotide segments, and indices were added to differentiate various samples within a single sequencing run.

Whole-genome sequencing was conducted using Illumina’s Next Generation Sequencing technology (Illumina, San Diego, CA, USA). The entire *E. coli* genome was sequenced using Illumina’s Miseq v2 Reagent Kit with 300 cycles (Illumina, CA, USA). Paired-end sequencing was performed to provide comprehensive genomic data.

### 3.7. Bioinformatic Analysis

The bioinformatics analysis was conducted using AMRViz, a toolkit developed by our research group that enables seamless genomics analysis and visualization of antimicrobial resistance [20]. AMRViz integrates a best practice analysis pipeline that automates the process from raw sequencing data to comprehensive genomic analysis.

The analysis included sequence quality assessment, cleaning, genome assembly, gene annotation, and profiling of antibiotic resistance and virulence genes. Sequence type (ST) determination and species identification were also performed. The pipeline allowed for the examination of both the core and accessory genome components, the translation of gene sequences into protein sequences, and subsequent gene alignment to identify protein variations and nucleotide mutations. Phylogenetic trees were constructed for each gene cluster to provide a detailed evolutionary analysis.

This comprehensive and high-throughput analysis pipeline was executed using Python 3.6 and adhered to standardized protocols available on GitHub (https://github.com/amromics/amrviz, accessed on 1 February 2024). By employing this rigorous bioinformatics approach, we ensured that the genomic data were meticulously examined, providing valuable insights into the genetic makeup of the *E. coli* strains, including their core and accessory genes, antibiotic resistance, and virulence gene profiles.

### 3.8. Data Analysis

The SPSS (version 21.0) software package for Windows (IBM Corp., Armonk, NY, USA) was employed for data analysis. Various statistical tests, including the Chi-square test and Fisher’s exact test, were applied to assess relationships and differences between variables. Specifically, the Chi-square test was used for larger datasets to determine the significance of associations between categorical variables. For smaller datasets (typically n < 5 in any cell of the contingency table), Fisher’s exact test was utilized due to its appropriateness for small sample sizes and ability to provide accurate significance levels. Statistical significance was determined when the *p*-value (statistical value) was less than 0.05, indicating the presence of a statistically significant relationship or difference.

## 4. Conclusions

The rising prevalence of drug-resistant Escherichia coli strains causing pediatric urinary tract infections (UTIs) underscores the urgent need for effective prevention and management strategies. This study leverages whole-genome sequencing to provide a comprehensive genetic characterization of *E. coli* strains, revealing significant temporal trends in antibiotic resistance genes in central Vietnam. Our findings highlight the increasing prevalence of key resistance genes, such as *aadA2* and *blaNDM-5*, which correlate with resistance to aminoglycosides and beta-lactams, including carbapenems. Additionally, there was a notable increase in the total number of antibiotic resistance genes per isolate over time. These insights emphasize the importance of continuous surveillance, targeted antibiotic stewardship, and the development of new therapeutic strategies to combat the evolving threat of antibiotic resistance in pediatric UTIs.

## Figures and Tables

**Table 1:** Characteristics of pediatric UTI cases and clinical *E. coli* isolates in central Vietnam.

Characteristic	Value
Epidemiological Characteristics	
- Isolation date Median (Oldest–Newest)	1 June 2019 (23 June 2018–27 October 2020)
- Gender Distribution (%)	
- Female	51.00%
- Male	49.00%
- Age Distribution (%)	
- 2 months to under 1 year	28.6%
- Other age groups	Varying
- Urban-Rural Distribution (%)	
- Rural	70.40%
- Urban	29.60%
Clinical Characteristics	
- Reasons for hospital admission	Fever, urinary symptoms, abdominal pain, others
- Body temperature range	Varied
- Urinary symptoms	Diverse
- External genitalia abnormalities	Documented
- Hematological and urinary abnormalities	White blood cell counts, neutrophil counts, C-reactive protein levels, urinalysis results
- Ultrasonography findings	Renal and bladder abnormalities
Microbiological analysis	
- Predominant causative agent	*E. coli* (83.7%)
- ESBL-Producing *E. coli* Isolates	60.9%
- Antibiotic resistance patterns	High resistance to Ampicillin (98.2%), Sulfamethoxazole/Trimethoprim (82.9%), Cephalosporin (68.6%), and variable resistance to other antibiotics

**Table 2:** Antibiotic resistance gene detection in *E. coli* isolates.

Index	Gene Names	Antibiotics Classes	Frequencies (%)
1	*blaEC*	Beta-lactams	70.21
2	*blaTEM-1*	Beta-lactams	51.06
3	*blaCTX-M-27*	Beta-lactams	51.06
4	*blaEC-5*	Betalactams	29.79
5	*blaDHA-1*	Beta-lactams	17.02
6	*blaNDM-5*	Beta-lactams	14.89
7	*blaCTX-M-55*	Beta-lactams	10.64
8	*blaCTX-M-15*	Beta-lactams	10.64
9	*blaOXA-1*	Beta-lactams	8.51
10	*blaCMY-42*	Beta-lactams	4.26
11	*blaCMY-2*	Beta-lactams	2.13
12	*aph(6)-Id*	Aminoglycoside	65.96
13	*aph(3″)-Ib*	Aminoglycoside	65.96
14	*aadA5*	Aminoglycoside	46.81
15	*aac(3)-IId*	Aminoglycoside	31.91
16	*aac(6′)-Ib-cr5*	Aminoglycoside and Quinolon	17.02
17	*aadA2*	Aminoglycoside	17.02
18	*rmtB1*	Aminoglycoside	12.77
19	*aac(3)-IIe*	Aminoglycoside	8.51
20	*aadA16*	Aminoglycoside	8.51
21	*aac(6′)-Ib-cr*	Aminoglycoside and Quinolon	2.13
22	*aadA1*	Aminoglycoside	2.13
23	*qnrB4*	Quinolon	17.02
24	*sul2*	Sulphonamide	80.85
25	*sul1*	Sulphonamide	65.96
26	*dfrA17*	Trimethoprim	59.57
27	*dfrA12*	Trimethoprim	17.02
28	*dfrA27*	Trimethoprim	10.64
29	*dfrA14*	Trimethoprim	6.38
30	*dfrA7*	Trimethoprim	6.38
31	*dfrA8*	Trimethoprim	2.13
32	*erm(B)*	Macrolid	82.98
33	*tet(A)*	Tetracyclin	61.70
34	*mph(A)*	MAC	44.68
35	*catA1*	Chloramphenicol	17.02
36	*ble*	Bleomycin	14.89
37	*arr-3*	Rifamycin	10.64
38	*catB3*	Chloramphenicol	8.51
39	*tet(B)*	Tetracyclin	6.38
40	*tet(D)*	Tetracyclin	2.13

## Data Availability

The datasets generated and/or analyzed during the current study are available in the NCBI’s BioProject, accessible via the accession number PRJNA1040333 (https://www.ncbi.nlm.nih.gov/bioproject/?term=PRJNA1040333, accessed on 9 July 2024).

## References

[1] Roberts K.B., Subcommittee on Urinary Tract Infection, Steering Committee on Quality Improvement and Management (2011). Urinary tract infection: Clinical practice guideline for the diagnosis and management of the initial UTI in febrile infants and children 2 to 24 months. Pediatrics.

[2] Mattoo T.K., Shaikh N., Nelson C.P. (2021). Contemporary Management of Urinary Tract Infection in Children. Pediatrics.

[3] Bryce A., Hay A.D., Lane I.F., Thornton H.V., Wootton M., Costelloe C. (2016). Global prevalence of antibiotic resistance in paediatric urinary tract infections caused by Escherichia coli and association with routine use of antibiotics in primary care: Systematic review and meta-analysis. BMJ.

[4] Prestinaci F., Pezzotti P., Pantosti A. (2015). Antimicrobial resistance: A global multifaceted phenomenon. Pathog. Glob. Health.

[5] Ha D.R., Haste N.M., Gluckstein D.P. (2019). The Role of Antibiotic Stewardship in Promoting Appropriate Antibiotic Use. Am. J. Lifestyle Med..

[6] Adamus-Bialek W., Baraniak A., Wawszczak M., Gluszek S., Gad B., Wrobel K., Bator P., Majchrzak M., Parniewski P. (2018). The genetic background of antibiotic resistance among clinical uropathogenic *Escherichia coli* strains. Mol. Biol. Rep..

[7] Tao S., Chen H., Li N., Wang T., Liang W. (2022). The Spread of Antibiotic Resistance Genes In Vivo Model. Can. J. Infect. Dis. Med. Microbiol..

[8] Dallman T.J., Byrne L., Ashton P.M., Cowley L.A., Perry N.T., Adak G., Petrovska L., Ellis R.J., Underwood A., Green J. (2015). Whole-genome sequencing for national surveillance of Shiga toxin-producing *Escherichia coli* O157. Clin. Infect. Dis..

[9] Moran-Gilad J., Rokney A., Danino D., Ferdous M., Alsana F., Baum M., Dukhan L., Agmon V., Anuka E., Valinsky L. (2017). Real-time genomic investigation underlying the public health response to a Shiga toxin-producing Escherichia coli O26:H11 outbreak in a nursery. Epidemiol. Infect..

[10] Quainoo S., Coolen J.P.M., van Hijum S.A.F.T., Huynen M.A., Melchers W.J.G., van Schaik W., Wertheim H.F.L. (2017). Whole-Genome Sequencing of Bacterial Pathogens: The Future of Nosocomial Outbreak Analysis. Clin. Microbiol. Rev..

[11] Zorc J.J., Kiddoo D.A., Shaw K.N. (2005). Diagnosis and management of pediatric urinary tract infections. Clin. Microbiol. Rev..

[12] Kaufman J., Temple-Smith M., Sanci L. (2019). Urinary tract infections in children: An overview of diagnosis and management. BMJ Paediatr. Open.

[13] Kwok W.Y., de Kwaadsteniet M.C., Harmsen M., van Suijlekom-Smit L.W., Schellevis F.G., van der Wouden J.C. (2006). Incidence rates and management of urinary tract infections among children in Dutch general practice: Results from a nation-wide registration study. BMC Pediatr..

[14] Rozwadowski M., Gawel D. (2022). Molecular Factors and Mechanisms Driving Multidrug Resistance in Uropathogenic *Escherichia coli*-An Update. Genes.

[15] Roberts L.W., Hoi L.T., A Khokhar F., Hoa N.T., Van Giang T., Bui C., Ninh T.H., Co D.X., Binh N.G., Long H.B. (2022). Genomic characterisation of multidrug-resistant *Escherichia coli*, *Klebsiella pneumoniae*, and *Acinetobacter baumannii* in two intensive care units in Hanoi, Viet Nam: A prospective observational cohort study. Lancet Microbe.

[16] Codjoe F.S., Donkor E.S. (2017). Carbapenem Resistance: A Review. Med. Sci..

[17] Ristuccia A.M., Cunha B.A. (1985). An overview of amikacin. Ther. Drug Monit..

[18] Jancel T., Dudas V. (2002). Management of uncomplicated urinary tract infections. West. J. Med..

[19] Mori R., Lakhanpaul M., Verrier-Jones K. (2007). Diagnosis and management of urinary tract infection in children: Summary of NICE guidance. BMJ.

[20] Le D.Q., Nguyen S.H., Nguyen T.T., Nguyen C.H., Ho T.H., Vo N.S., Nguyen T., Nguyen H.A., Cao M.D. (2024). AMRViz enables seamless genomics analysis and visualization of antimicrobial resistance. BMC Bioinform..

